# The powerful world of antisense oligonucleotides: From bench to bedside

**DOI:** 10.1002/wrna.1594

**Published:** 2020-03-31

**Authors:** Anaïs M. Quemener, Laura Bachelot, Anne Forestier, Emmanuelle Donnou‐Fournet, David Gilot, Marie‐Dominique Galibert

**Affiliations:** ^1^ Univ Rennes, CNRS, IGDR (Institut de Génétique et Développement de Rennes)—UMR6290, ARC Foundation Labellized Team Rennes France; ^2^ Department of Molecular Genetics and Genomic CHU Rennes, Hospital‐University of Rennes Rennes France

**Keywords:** antisense oligonucleotide, ASO‐therapy, cancer, genetic disoders, miRNA sponge

## Abstract

Antisense oligonucleotides (ASOs) represent a new and highly promising class of drugs for personalized medicine. In the last decade, major chemical developments and improvements of the backbone structure of ASOs have transformed them into true approved and commercialized drugs. ASOs target both DNA and RNA, including pre‐mRNA, mRNA, and ncRDA, based on sequence complementary. They are designed to be specific for each identified molecular and genetic alteration to restore a normal, physiological situation. Thus, the characterization of the underpinning mechanisms and alterations that sustain pathology is critical for accurate ASO‐design. ASOs can be used to cure both rare and common diseases, such as orphan genetic alterations and cancer. Through pioneering examples, this review shows the versatility of the mechanisms of action that provide ASOs with the potential capacity to achieve custom treatment, revolutionizing personalized medicine.

This article is categorized under:

RNA in Disease and Development > RNA in Disease
RNA Interactions with Proteins and Other Molecules > Small Molecule–RNA Interactions

## Graphical abstract

ASO functioning towards personalized medicine.

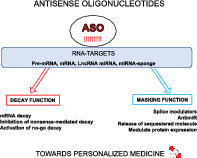

## 1 INTRODUCTION

Recent improvements in pharmacology, together with an understanding of the molecular mechanisms underlying diseases, have led to significant advances in the therapeutic management of patients. Personalized and precision medicine is now becoming essential, alongside more general and sometimes only symptomatic treatment. Personalized medicine is adapted to the genetic, molecular, and phenotypic characteristics of the disease and the patient, by targeting specifically causative components. Importantly, one of the major challenges in the current management of many diseases is predicting the responses to treatment as a function of patient‐intrinsic characteristics. There is, therefore, a genuine revolution underway in patient care.

The understanding of biological mechanisms has made possible the development of the first targeted therapies. They were initially directed against the proteins responsible for, or specifically associated with, a disease. The development of hormonal treatments for so‐called “hormone‐dependent” breast cancers (ER‐positive, estrogen receptor‐positive) was driven by the understanding of the role of ER in breast cancer and the identification of mechanisms for its blockade. Tamoxifen is now a standard treatment for ER‐positive breast cancers. It acts by competitively inhibiting the binding of estradiol to its receptors (Jordan, 2003). Monoclonal antibodies directed against defined epitopes also constitute a very important class of targeted therapies. They revolutionized the management of inflammatory diseases, such as asthma (Pelaia et al., 2017). However, the identification of the genetic alterations responsible for diseases has provided the major impetus for the use of targeted therapies. For example, the reciprocal translocation t(9; 22), known as the Philadelphia chromosome, is a hallmark of chronic myeloid leukemia (CML). The t(9;22) translocation was, therefore, first used to confirm the diagnosis of CML (Heisterkamp et al., 1990; Rowley, 1973). This translocation generates an aberrant fusion gene (*BCR‐ABL*). The resulting BCR‐ABL fusion protein has oncogenic properties due to its constitutive tyrosine kinase activity (Lugo, Pendergast, Muller, & Witte, 1990). The development of a competitive inhibitor of ATP binding to the catalytic site of the protein kinase led to a specific therapy: imatinib or Gleevec®, revolutionizing the management of CML and other diseases (Kantarjian & Talpaz, 2001). Similarly, the identification of oncogenic *NTRK* (Neurotrophic Tropomyosin Related Kinase) fusion genes recently led to the development of specific inhibitors (larotrectinib or Vitrakvi®, entrectinib or Rozlytrek®) for the management of NTRK‐positive cancers in adults and children (Cocco, Scaltriti, & Drilon, 2018). In oncology, specific inhibitors targeting recurrent point mutations have also been widely developed (Martini, Vecchione, Siena, Tejpar, & Bardelli, 2012; Skoulidis & Heymach, 2019).

In some cases, very little or no protein is produced. This is true for insulin, an enzyme that is deficient in patients with insulin‐dependent diabetes (type I). Patients are treated by insulin therapy to faithfully reproduce the effects of the physiological secretion of insulin by administering a substitute protein. The release of the first human insulin protein onto the market in 1982 launched a new paradigm: it became possible to modify the sequence of the hormone protein to match the pharmacokinetic properties to the physiological needs of the patient (McCall & Farhy, 2013).

In addition to these “protein‐specific” therapies, approaches targeting DNA (deoxyribonucleic acid) have been developed. As for proteins, the first therapeutic attempts were based on global alterations to DNA, through the use of alkylating agents, for example. These agents induce the creation of non‐specific covalent bonds, resulting in the production of DNA‐adducts. They disrupt replication and transcription, explaining their use in cancer therapeutics (Noll, Mason, & Miller, 2006). Intercalation is also a special binding mode for small planar molecules to DNA. They alter the conformation of the DNA, disrupting the activities of DNA and RNA polymerases (Binaschi, Zunino, & Capranico, 1995). The use of DNA‐targeting molecules is not limited to oncological applications. For example, methotrexate, an antimetabolite that inhibits nucleic‐acid synthesis during the S phase of the cell cycle, has replaced the traditionally used silver salts in the treatment of rheumatoid arthritis (Browning, Rice, Lee, & Baker, 1947). In parallel with these molecules that interact with DNA in a non‐specific manner, targeted strategies correcting a deleterious gene responsible for a disease have been conceived. This approach is known as gene therapy (Kaufmann, Büning, Galy, Schambach, & Grez, 2013). One very promising example (marketing authorization [MA] pending) concerns the treatment of β‐thalassemia, a genetic disorder of hemoglobin. Here, the patient's stem cells are isolated and modified to replace the deleterious gene, such that they can produce normal hemoglobin. The modified cells are then injected back into the patient (Cavazzana‐Calvo et al., 2010; Thompson et al., 2018). These spectacular approaches could be considered for many diseases, including diabetes, although they are very complex to put in place.

**Figure 1:**
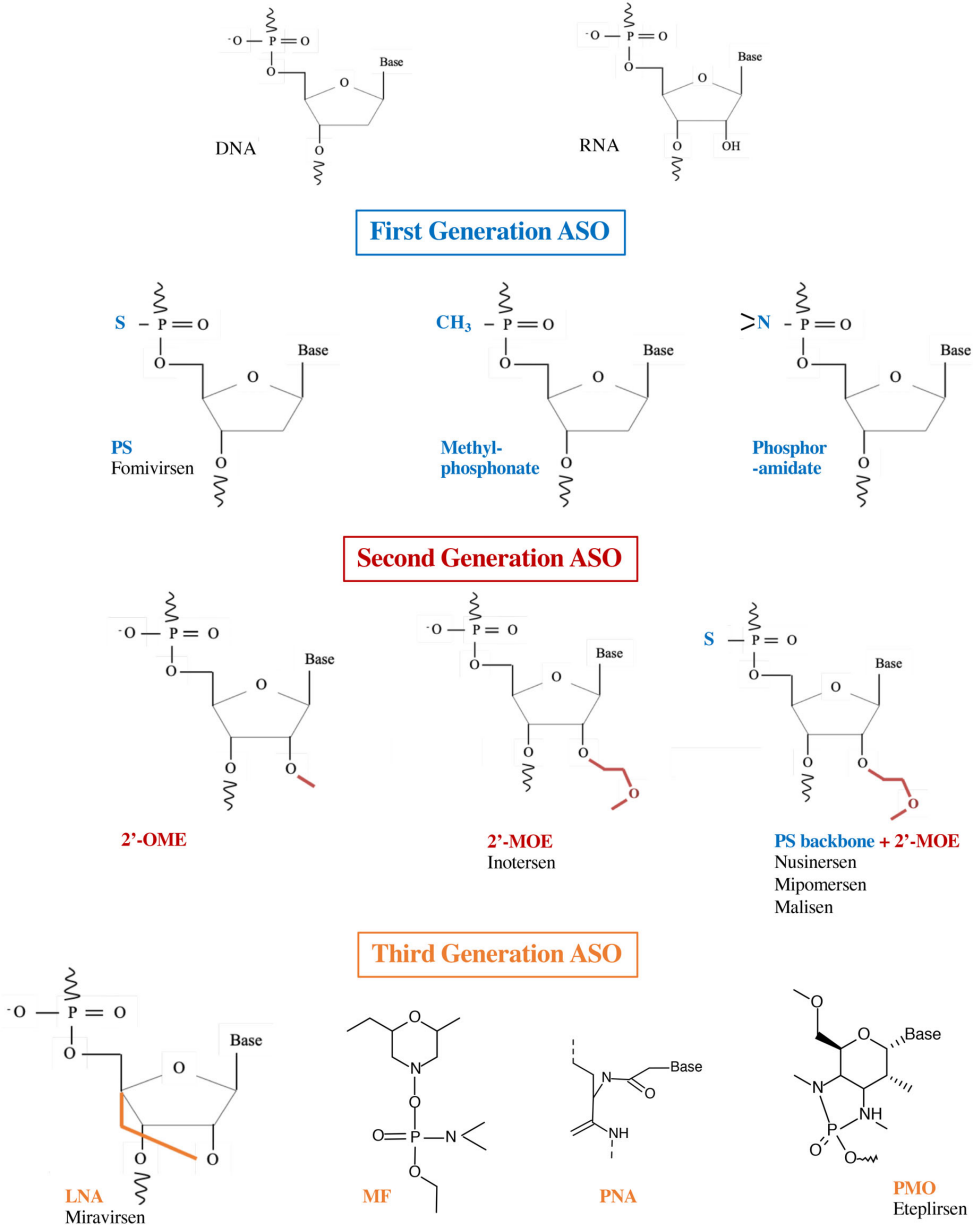
Chemical structure of first, second, and third generation ASOs, compared to DNA and RNA structures, followed with examples of FDA‐approved drugs. 2′‐MOE, 2′‐*O*‐methoxyethyl; 2′‐OME, 2‐*O*‐methyl; ASOs, antisense oligonucleotides; DNA, deoxyribonucleotidic acid; LNA, locked nucleic acid; MF, morpholino phosphorodiamidate; PMO, phosphorodiamidate morpholino oligomer; PNA, peptide nucleic acid; PS, phosphorothioate; RNA, ribonucleotidic acid

**Figure 2:**
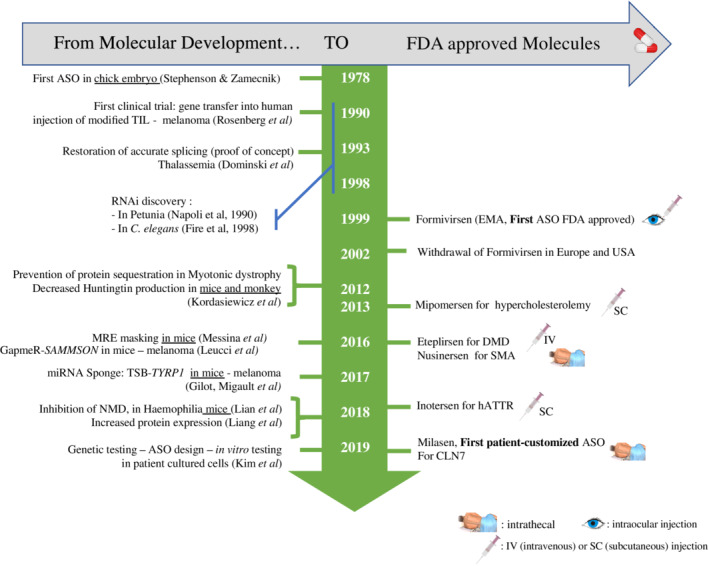
Timeline of ASO drug development and approval. ASO, antisense oligonucleotide; hATTR, polyneuropathy of hereditary transthyretin‐mediated amyloidosis; NMD, nonsense‐mediated decay; SMA, spinal muscular amyotrophy

Finally, mRNA, which was long considered to be a simple intermediate molecule, has recently become a therapeutic target of interest. mRNA is the site of both fine transcriptional and post‐transcriptional regulation, being involved in many diseases. As a result, attention has also focused on RNA molecules in recent years, because these molecules, like proteins and DNA, are candidates of interest for the development of targeted therapies (Disney, Dwyer, & Childs‐Disney, 2018). The first antisense oligonucleotides (ASOs) emerged in this context. ASOs are single‐stranded synthetic RNA or DNA molecules with a mean length of 12 to 25 nucleotides. Their sequences are complementary to that of their target, to ensure specificity. The sequence of the ASO is, therefore, dictated by the sequence of its target. Moreover, these molecules can be localized in both the cytoplasm and nucleus, making it possible to reach cytoplasmic and/or nuclear targets (reviewed in Potaczek, Garn, Unger, & Renz, 2016). ASOs are chemically modified to protect them against the action of nucleases that would otherwise degrade them, and to allow them to pass through the plasma membrane without the need for vectorization. ASOs can be classified into three main generations (described below) on the basis of these changes (Figure 1). The chemistry of the ASO is important because it determines its mode of action (degradation of the target RNA or masking a site without degradation). ASOs can, therefore, be extensively modulated, making them ideal for use in targeted therapy. Zamecnik and Stephenson first used ASOs for therapeutic purposes in 1978. They used a 13‐nucleotide DNA molecule complementary to the Rous sarcoma virus ribosomal RNA to inhibit its translation, thereby preventing the oncogenic transformation of chicken fibroblasts infected with Rous virus in vitro (Figure 2) (Stephenson & Zamecnik, 1978).

Several molecules have now received marketing authorization from drug agencies (Food and Drug Administration (FDA) in the United States of America and European Medicines Agency (EMA) in Europe). This is the result of many years of basic research and clinical trials. Throughout pioneering examples, this review aims to underscore the versatility of ASOs for the treatment of various diseases through the targeted and personalized RNA‐based approach they allow.

### 1.1 What type of chemistry for ASOs?

ASOs are short (about 12 to 25 nucleotides long) single‐stranded nucleic acid (DNA or RNA) sequences. In their naked form, they cannot permeate the plasma membrane and are highly sensitive to degradation by endonucleases and exonucleases. ASOs have been chemically modified to overcome these problems. On the basis of these modifications, ASOs can be broadly classified into three generations (Mansoor & Melendez, 2008).

In first‐generation ASOs, the phosphate backbone linking the nucleotides is modified. One of the non‐bridging oxygen atoms in the phosphodiester bond is replaced by a sulfur, methyl or amine group, generating phosphorothioates (PS), methyl‐phosphonates, and phosphoramidates, respectively (Figure 1). These modifications are not equivalent, and each has its own specific features. PS oligonucleotides are highly representative of this first generation, being the most widely used. These chemical modifications improve stability by increasing the resistance of ASOs to nucleases, a constant goal being to expand the ASO half‐life. PS modifications transform the half‐life from minutes to days. Importantly, these modifications activate RNAse H (Campbell, Bacon, & Wickstrom, 1990; Kurreck, 2003; Stein, Subasinghe, Shinozuka, & Cohen, 1988). RNAse H is a ubiquitously expressed enzyme that cleaves the RNA strand in a DNA–RNA duplex (Furdon, Dominski, & Kole, 1989; Miller, Riggs, & Gill, 1973). RNAse H can, therefore, degrade the target mRNA within the ASO/mRNA complexes, limiting the synthesis of the encoded protein. Unfortunately, the biologically active modified ASOs (PS) are highly toxic due, in particular, to their non‐specific binding to proteins (Koziolkiewicz et al., 2001). This led researchers to develop new generations of ASOs that were both less toxic and more specific.

The second generation of ASOs is characterized by alkyl modifications at the 2′ position of the ribose. The introduction of an oxygenated group leads to the formation of 2′‐*O*‐methyl (2′‐OME) and 2′‐*O*‐methoxyethyl (2′‐MOE) nucleotides (Figure 1) (Monia et al., 1993; Nicolussi, D'Inzeo, Capalbo, Giannini, & Coppa, 2017). These ASOs are less toxic than PS and have a slightly higher affinity for their target. However, these modifications are incompatible with the recruitment of and cleavage by RNAse H. The antisense effect of this type of ASO is probably due to steric blockade of translation. Such modifications are of potential interest if the target RNA must not be degraded.

The third generation is more heterogeneous because it includes a large number of modifications aiming to improve binding‐affinity, resistance to nucleases, and pharmacokinetic profile (Figure 1) (Khvorova & Watts, 2017). The most common modifications include locked nucleic acids (LNAs), corresponding to a methylene bridge connecting the 2′‐oxygen and 4′‐carbon of the ribose (Hagedorn et al., 2018); phosphorodiamidate morpholino oligomers (PMOs), in which the ribose is replaced by a morpholine moiety and the phosphodiester bond by a phosphorodiamidate bond; and peptide nucleic acids (PNAs), in which the ribose‐phosphate backbone is replaced by a polyamide backbone consisting of repeats of N‐(2‐aminothyl) glycine units, to which the bases are linked (Egholm et al., 1993; Nielsen, Egholm, Berg, & Buchardt, 1991). These last two structures are uncharged and bind to plasma proteins with a lower affinity than charged ASOs, which increases their distribution and elimination in urine (Shen et al., 2019). The fraction eliminated has been shown to correspond to approximately 10–30% of the amount administered, contributing to tissue accumulation (Amantana & Iversen, 2005). These modifications confer high stability but do not elicit RNAse H recruitment. This third generation of ASO forms a stable hybrid with its target mRNA, thereby interfering with its processing or translation.

The conformational constraint of the LNA modification imposed by the connecting bridge and that of its methylated analog (known as “constrained ethyl”: cET) have created new opportunities in chemical therapeutics. Tricyclo‐DNA (tcDNA) belongs to this class of conformationally constrained DNA analogs with enhanced binding properties. They do not elicit RNAse‐H activity but show increased stability and improved cellular uptake, giving them substantial therapeutic advantages over those of ASOs (Goyenvalle et al., 2015; Renneberg, Bouliong, Reber, Schümperli, & Leumann, 2002; Steffens & Leumann, 1997).

As underscored previously, ASOs carrying most second‐ and third‐generation chemical modifications do not elicit RNAse H activity. RNAse H activity can however be restored by inserting a stretch of unmodified or PS‐DNA cleavage‐sensitive sequence between a pair of non‐RNAse H‐sensitive sequences at the ends of the ASO. The resulting structure is known as a “gapmer.” The “gapmer” approach was first shown by Inoue for a 2′OME PO ASO (Inoue et al., 1987), followed by a 2′MOE gapmer in 1993 (Monia et al., 1993). Nearly a decade later, the gapmer structure was used with a third generation ASO (Kurreck, 2003; Shen, Kandimalla, & Agrawal, 1998; Veedu & Wengel, 2009).

Overall, such diversity of chemical modifications, together with the structure of the ASO, offers considerable flexibility for the adaptation of the therapeutic approach according to the chosen target and mechanism of action.

### 1.2 One or several mechanisms of action?

The working principle of ASO depends on its intrinsic parameters (RNA or DNA sequence and chemistry) and the target sequence. The choice of ASO is, therefore, guided by the required molecular effect (reviewed in Kurreck, 2003; Potaczek et al., 2016).

#### 1.2.1 Degradation of the mRNA to decrease protein levels

Levels of mRNA are tightly regulated by the binding of small non‐coding RNAs, microRNAs (miRNAs), by sequence complementarity. This process was first described in plants (Napoli, Lemieux, & Jorgensen, 1990) and then in the nematode *Caenorhabditis elegans* (Fire et al., 1998). These discoveries opened up a new field, that of RNA interference (Mello & Conte, 2004). In addition to improving our fundamental knowledge, these discoveries have increased our understanding of certain diseases and made it possible to develop new therapeutic strategies. The regulation exerted by endogenous miRNAs can be mimicked by exogenous molecules, such as ASOs. The binding of these molecules to a target can illicit its degradation.

**Figure 3:**
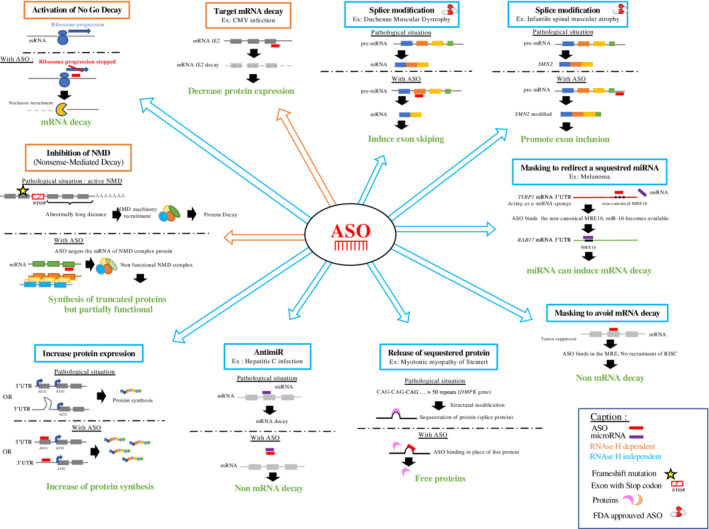
Schematic representation of multiple therapeutic options using ASOs. ASOs, antisense oligonucleotides

This strategy led to the development of the first ASO (fomivirsen) approved by the FDA in 1998 and the EMA in 1999 (Figure 2). Fomivirsen was marketed by Ionis Pharmaceuticals (formerly Isis) and Novartis Ophthalmics under the name Vitravene® (or Isis 2,922). Fomivirsen is a phosphorothioate oligodeoxynucleotide, the only first‐generation ASO to have received marketing authorization. It was used to treat cytomegalovirus (CMV) retinitis. This ASO specifically targets the viral mRNA encoding the immediate early‐2 (IE‐2) protein. IE‐2 is required for viral replication (Figure 3). In healthy individuals, CMV infection often goes unnoticed. By contrast, infection may be severe in immunodeficient individuals. This treatment was, therefore, recommended for patients with acquired immunodeficiency syndrome (AIDS), in whom CMV infection can lead to the progressive destruction of retinal cells (Geary, Henry, & Grillone, 2002; Roehr, 1998; Vitravene Study Group, 2002). Given the localized nature of the cells to be treated, local administration was approved. Fomivirsen was injected intraocularly (in the vitreous humor) at a dose of 165 μg per week for 3 weeks, in newly diagnosed patients. The dose was doubled for patients diagnosed at an advanced stage. This targeted therapy was effective, but caused severe localized adverse effects (increased intraocular pressure and local inflammation) (Perry & Barman Balfour, 1999). Fomivirsen was withdrawn from the market in 2002 in Europe and 2006 in the US (EMA: EMEA/12382/02; FDA document number: 2011‐14164) (Figure 2). Fortunately, the tri‐therapy now used to treat AIDS has substantially decreased the prevalence of CMV infection. The withdrawal of fomivirsen has not hindered the development of new ASOs but has rather fostered the development of less toxic molecules.

#### 1.2.2 ASOs for modifying or correcting splicing events

Maturation of pre‐mRNA into mRNA is a normal process in which intron sequences are excluded and exons are selected to form the mature mRNA. This process is tightly regulated, making it possible to generate several protein isoforms from the same pre‐mRNA, depending on the cellular context. Isoforms may have very similar functions or they may be very different. They can be ubiquitous or have a cell/organ‐specific location. Splicing is not, therefore, random and involves a large number of proteins forming a splicing complex, which specifically recognizes the splice donor and acceptor sites (Matera & Wang, 2014). Splicing defects can cause severe illness. In 1993, Dominski and Kole demonstrated that ASOs, here called splice switchers, could restore splice defects in patients with *β*‐thalassemia. They showed that 2′‐*O*‐methylribooligonucleotides targeting specific sequence elements in mutated human *β*‐globin pre‐mRNA could eliminate the aberrant splicing pattern of the *β*‐globin pre‐mRNA (Figure 2) (Dominski & Kole, 1993). These results served as a proof‐of‐concept and opened up new therapeutic perspectives, with the ability to modulate or block the splicing machinery. This strategy has been successful in the treatment of spinal muscular atrophy (SMA). SMA is an autosomal recessive neuromuscular disease caused by the progressive loss of motor neurons, leading to muscle atrophy and infant death. SMA is due to deleterious mutations of the *SMN1* (survival of motor neuron 1) gene. These genetic alterations lead to the absence of SMN protein production. Interestingly, SMN proteins are encoded by two paralogous genes: *SMN1* and *SMN2*. The *SMN1* and *SMN2* genes are identical (99% homology), except for an 11‐nucleotide sequence in exon 7. This difference alters splicing. The splicing of the *SMN2* pre‐mRNA leads to the exclusion of exon 7, generating a truncated, unstable protein that is rapidly degraded. *SMN2* cannot, therefore, compensate for the loss of *SMN1* expression in SMA. Nusinersen, discovered by Ionis Pharmaceuticals and co‐developed by Biogen (Spinraza®), was designed to correct *SMN2* splicing, thus allowing *SMN2* to compensate for the loss of expression caused by the *SMN1* mutation. This 2′‐*O*‐methoxyethyl (2′‐OME) phosphorothioate (PS) 18‐mer is a second‐generation ASO, which cannot recruit RNAse H. It targets the intronic splicing silencer (ISS‐N1), promoting the inclusion of exon 7, thereby restoring the production of a stable SMN2 protein (Figure 2) (Hua et al., 2010; Hua et al., 2011; Mercuri et al., 2018). Clinical trials proved its efficacy, leading to FDA and EMA approval in 2016. Nusinersen is administered intrathecally on weeks 0, 2, 4, and 8, followed by an injection once every 4 months for maintenance (Figures 2 & 3) (Biogen, 2019; Finkel et al., 2017; Mercuri et al., 2018).

#### 1.2.3 Masking of miRNA response element (MRE) sites

ASOs can be used to mask miRNA‐binding sites of mRNAs to modulate their expression. MicroRNAs are small (mean length: 22 nucleotides) non‐coding RNAs that specifically hybridize to mRNAs to fine‐tune their expression. miRNAs bind to their cognate target RNA through RNA–RNA base‐pairing. The miRNA binding sequence is referred to as a miRNA recognition element (MRE). MREs are complementary to miRNA seed regions (Bartel, 2009). Perfect base‐pairing leads to the recruitment of a protein complex, resulting in degradation of the target mRNA (Song, Smith, Hannon, & Joshua‐Tor, 2004).

The overexpression of miRNA in certain diseases, including cancers, can lead to massive pathological degradation of their targets. ASOs binding to specific MREs could thus be used to block such mRNA degradation. In this case, ASOs are specific for mRNA and not miRNA. No therapeutic molecules based on this mode of action are currently available. However, the group of Prevot has demonstrated the efficacy of such an approach (Messina et al., 2016). Their studies focus on the role of miRNAs in the production of pituitary gonadotropins (GnRH) during development, especially before puberty. In this context, third‐generation ASOs, mixmers composed of modified nucleotides (LNAs), marketed as target‐site blockers (TSB), have been used to validate the targets of certain miRNAs. In general, TSBs bind to RNA without inducing RNAse H activity. Their sequences are 100% complementary to those of the RNA sequence containing the MRE motif, resulting in specific binding to the RNA target. As TSBs have a higher (100% complementary) affinity than miRNAs for the same MRE sequence, competitive binding favors the ASO (Figure 3). In vivo, TSBs increase the expression of their specific targets, without affecting other targets of the same miRNA. Interestingly, the TSBs were injected directly into mouse brains (Messina et al., 2016).

#### 1.2.4 Release of a sequestered protein

The role of mRNAs is not necessarily restricted to coding; some have structural functions. These functions may be physiological or pathological and can, therefore, be targeted by treatments.

Steinert's disease, also known as type 1 myotonic dystrophy (DM1), is a clinically heterogeneous neuromuscular disease. DM1 is a triplet‐repeat disease originating from progressive expansion of CTG repeats in the 3′ UTR of the *DMPK* (DM1 protein kinase) gene (Orengo et al., 2008). The disease displays autosomal dominant transmission, and disease severity varies with the number of CTG repeats (Brook et al., 1992). The excess of triplet‐repeats results in the formation of secondary stem‐loop structures on the mRNA, which does not exit the nucleus. These toxic mRNA molecules accumulate in the nucleus and their secondary structures associate with proteins, such as MBNL1 (muscleblind‐like splicing regulator 1). As MBNL1 protein is involved in the regulation of splicing, its sequestration in nuclear foci leads to multiple splicing abnormalities and spliceopathies. ASOs are being studied as possible replacements for the chemical inhibitors used to treat this disease (Haghighat Jahromi, Honda, Zimmerman, & Spies, 2013). Several strategies are possible.

The first involves degrading the toxic mRNA to release the sequestered protein. By degrading the *DMPK* mRNA with an ASO displaying RNAse H activity (MOE gapmers), the sequestered MBNL1 protein is released and accurate splicing is restored (Langlois, Lee, Rossi, & Puymirat, 2003; Mulders et al., 2009). This strategy is illustrated in Figure 3 (Wheeler et al., 2012).

The second strategy is based on competitive binding between ASOs and the sequestered protein. Here, a CAG‐repeat morpholino oligomer (without activating RNAse H) competes with MBNL binding to the CUG‐repeats. This ASO has been shown to be effective in triggering the release of MBNL and restoring splicing function in mice (Wheeler et al., 2012).

#### 1.2.5 Modulation of RNA translation quality‐control mechanisms

Multiple quality control mechanisms recognize and eliminate defective mRNA molecules during the process of translation to ensure RNA and protein quality.

Nonsense‐mediated decay (NMD) is one of such quality‐control mechanisms for mRNAs. It eliminates mRNAs that include a premature stop codon. This allows the removal of abnormal proteins that are non‐functional or with impaired function, and prevents the production of proteins with dominant‐negative activity (Frischmeyer & Dietz, 1999). However, in certain situations, the maintenance of partially‐functional truncated proteins may be of clinical interest. In these particular situations, therapeutic approaches targeting NMD may be attractive. NMD inhibition must be tightly controlled and adapted, to prevent dramatic consequences. The implementation of such approaches requires a thorough knowledge of the various components of the NMD complex to specifically disrupt only one of them. Huang et al. recently identified several candidate factors, including the Upf3b regulator of NMD, for the development of a specific ASO (L. Huang et al., 2018). Consistent with previous results, they showed that Upf3b is not essential for development and that it regulates only a subset of endogenous NMD substrates. Upf3b depletion is well tolerated, with minimal impact on normal transcriptome. Upf3b‐ASO treatment resulted in significant stabilization of the *dystrophin* mRNA in MDX mice, a model of Duchenne myopathy that harbors a nonsense mutation (CAA to TAA) in exon 23 of the dystrophin gene, resulting in a premature translation termination codon. They obtained comparable results in a hemophilia mouse model, in which Upf3b‐ASO treatment restored the production of functional factor IX protein (Figure 3) (Huang et al., 2018; Zhang et al., 2019).

This approach has been studied only in mice, but shows much promise. This strategy is less precise than other modes of action, because it acts on a ubiquitous regulatory pathway. This shows that ASOs can also be used for more global strategies.

Another mechanism involved in translational control is no‐go decay (NGD). NGD is triggered by obstacles that impede ribosome movement along the mRNA. These altered mRNA molecules are targeted for degradation, initiated by endonucleolytic cleavage in the vicinity of the stalled ribosome (Harigaya & Parker, 2010). Comparable to natural obstacles, ASOs that do not initiate RNAse H cleavage can be used to bind to the coding sequence and disturb ribosome scanning, leading to stalling of the ribosome. In this specific context, ASOs can promote the NGD pathway, leading to a decrease of the target mRNA. This mechanism has been demonstrated by Liang et al. in several human and mouse cell lines Figure 3 (Liang, Nichols, Hsu, Vickers, & Crooke, 2019). This original ASO‐driven approach constitutes a promising alternative option to induce mRNA decay.

#### 1.2.6 Increasing protein levels

Certain diseases are characterized by a deficit in the synthesis of a given protein; specifically increasing its production is, therefore, of potential therapeutic interest. Although still very challenging, efforts are being made to increase the production of selected proteins using ASOs. The group of Crooke has used modified ASOs that bind to mRNA sequences in the upstream open reading frame (uORF) to specifically increase the synthesis of the main protein translated from a downstream primary ORF (pORF). They generated ASOs (2‐*O*‐methyl modifications and phosphodiester [PO] links) that specifically hybridize to the start codon (uAUG) of such uORFs. By specifically blocking translation of the uORF, the ASO redirects the translation machinery to the pORF. The efficacy and specificity of this approach has been evaluated with several human and mouse model genes (including the human *RNAseH1* and sideroflexin 3 [*SFXN3*] genes). This resulted in a significant increase in the level of the corresponding proteins (Liang et al., 2016).

They also showed that ASOs can specifically bind to the secondary structures of 5′ UTRs, shown to be negative regulators of translation. Various ASOs designed to bind to these loop‐stem structures can increase translation. The human *RNAseH1*, *LDLR*, and *ACP1* and mouse *ARF1* and *ACP1* genes have been targeted, resulting in significant increases in the levels of the corresponding proteins (Liang et al., 2017). These two approaches are complementary, as not all mRNAs have an upstream start codon or secondary structures (Figure 3).

In parallel to these strategies that directly target regulatory sequences, it is possible to increase the level of a protein through indirect routes. Such a strategy has been used to increase the levels of the low‐density lipoprotein receptor (LDLR) by targeting its regulator PSCK9 (Gupta et al., 2010). The proprotein PCSK9 (protein convertase subtilisin/kexin type 9) is a key etiological factor in familial hypercholesterolemia. PCSK9 accelerates LDLR degradation (Cohen, Boerwinkle, Mosley, & Hobbs, 2006). Low levels of hepatic PCSK9 are, therefore, associated with higher LDLR levels, faster clearance of LDL‐cholesterol, and lower levels of LDL‐cholesterol in the bloodstream. The use of ASO‐LNA specifically targeting the *PCSK9* transcript leads to a sharp decrease of PCSK9 levels in vitro (liver cells) and in vivo (mouse liver) (Gupta et al., 2010). Although not an ASO, Inclisiran, a siRNA targeting *PCSK9* mRNA, is currently being tested in preclinical trials (ORION‐1, ClinicalTrials.gov number: NCT03705234) (Ray et al., 2017). It acts by a different mechanism. Once the double‐stranded siRNA enters the cell, the complementary strand of the target or guide strand is loaded onto the RISC complex (RNA‐induced silencing complex). The target RNA is specifically recognized by complementary hybridization with the siRNA and cleaved by one of the proteins of the RISC complex (Figure 3). Inclisiran has been chemically modified to increase its stability and duration of action (Fitzgerald et al., 2017).

#### 1.2.7 Targeting pathological miRNAs: Anti‐miRs

MicroRNAs tightly regulate gene expression, thereby controlling many physiological functions. Because they are important regulators, they are also associated with disease. Inhibition of their activity may, therefore, be an effective therapeutic strategy (Krützfeldt et al., 2005). Anti‐miRs are ASOs with sequence complementary to the endogenous miRNA targeted, forming stable, high‐affinity bonds. Like ASOs, they can be synthesized with various chemical characteristics (Stenvang, Petri, Lindow, Obad, & Kauppinen, 2012).

Miravirsen (Roche/Santaris) is one of the first miRNA therapies to be developed and undergo clinical testing. It targets miR‐122 for the treatment of hepatitis C virus infections. miR‐122 is a key player in liver development. This abundant miRNA is produced in a specific manner. It also plays an important role in the replication cycle of the hepatitis C virus (HCV), helping to stabilize the viral genome. Patients infected with HCV have a long‐term risk of developing liver cancer (Lee, Yang, Yuan, L'Italien, & Chen, 2014). The sequestration of miR‐122 with an anti‐miR is, therefore, of therapeutic interest. Miravirsen is a third‐generation ASO (ASO‐LNA). Its sequence is perfectly complementary to that of the mature miR‐122, and its binding to this miRNA blocks its interactions with its targets, including HCV‐RNA. No effect on the plasma levels of other miRNAs has been recorded. This ASO is also complementary to the precursors of miR‐122 (pre‐miR122 and pri‐miR‐122). It therefore blocks the biogenesis of miR‐122, reducing its production, as it prevents dicer and drosha, key enzymes in miRNA biogenesis, from acting (Figure 2). Its administration to HCV‐infected primates effectively decreases the viral RNA load (Lanford et al., 2010). Phase IIa trials in humans have also reported a decrease in viral RNA levels, but the RNA does not completely disappear (Janssen et al., 2013).

The potential value of the anti‐miR approach probably lies in the weak induction of resistance, which has led to current trials testing miravirsen in combination with powerful antiviral drugs that would normally favor the emergence of resistance (Liu et al., 2016).

The obvious therapeutic value of this molecule led to the development of RG‐101 (Regulus Therapeutics). Like miravirsen, RG101 is an anti‐miR‐122 agent. It differs from miravirsen in its chemical structure. It is an ASO phosphorothioate coupled to an N‐acetylgalactosamine group (GalNac), to enhance uptake by hepatocytes via the asialoglycoprotein receptor. Despite these significant improvements and the therapeutic effects observed (Van Der Ree et al., 2016), Regulus halted the development of the molecule in 2017, due, in particular, to its liver toxicity (increase in bilirubin levels). Despite these setbacks, there is still genuine enthusiasm for the development of anti‐miRs, as demonstrated by the clinical trials currently underway (Figure 3) (reviewed in Chakraborty, Sharma, Sharma, Doss, & Lee, 2017).

### 1.3 What can we target with ASOs?

Regardless of the mode of action, the target molecule is a coding or non‐coding RNA.

#### 1.3.1 Coding RNAs

As previously described, ASOs may target coding RNAs through various modes of action. This strategy has been used for the treatment of familial homozygous hypercholesterolemia. Familial homozygous hypercholesterolemia is a rare genetic disease resulting in high concentrations of atherogenic LDL particles, which are involved in cholesterol transport. Mipomersen (Kynamro®) is an inhibitor of the synthesis of ApoB100 (Raal et al., 2010), the main component of LDLs and their precursors, VLDLs. The use of a 20‐nucleotide 2′‐*O*‐methoxyethyl‐modified phosphorothioate oligonucleotide (second‐generation ASO) complementary to the coding region of the *ApoB100* mRNA leads to its degradation by RNAse H. Mipomersen use over several weeks induces a progressive decrease of LDL‐cholesterol levels (Akdim et al., 2011; Ricotta & Frishman, 2012). Mipomersen was developed and marketed by Ionis Pharmaceuticals, in collaboration with Genzyme Corporation. Following the demonstration of its efficacy in clinical trials, with only moderate adverse effects (influenza‐like illness and skin reaction at the injection site), it was approved by the FDA in the United States in 2013 for the treatment of familial hypercholesterolemia in adults (Figure 2). Mipomersen is administered subcutaneously once per week (200 mg/week), and direct self‐administration by the patient is possible. It is used in combination with other cholesterol‐lowering drugs to improve treatment efficacy (Figure 3) (Akdim et al., 2011; Hair, Cameron, & McKeage, 2013; Santos et al., 2015).

#### 1.3.2 Non‐coding RNAs

ASOs can also target non‐coding RNAs (long non‐coding RNA [lncRNA], miRNA, etc.), which, like coding RNAs, may cause disease. Angelman syndrome is a genetic neurological disorder characterized by severe motor and intellectual disability (Clayton‐Smith & Laan, 2003). Several causal molecular abnormalities have been identified, all of which involve a lack of function of part of the maternal chromosome 15 (15q11‐q13 region). The *UBE3A* gene is located in this region and plays a central role. When the *UBE3A* gene inherited from the mother carries a deleterious mutation or is not expressed, the patient develops Angelman syndrome, despite the presence of a normal but inactivated paternal copy. Releasing the inactivation of the paternal copy of the *UBE3A* gene constitute one possible treatment. Such inactivation is controlled by a lncRNA, *UBE3A‐ATS* (UBE3A‐antisense) (Mabb, Judson, Zylka, & Philpot, 2011; Rabinovitz, Kaufman, Ludwig, Razin, & Shemer, 2012). Meng et al. developed an ASO targeting *UBE3A‐ATS* to restore the expression and activity of UBE3A. They used this approach in a mouse model and showed that a second‐generation ASO (2′‐MOE) complementary to the non‐coding *UBEA3‐ATS* RNA caused its degradation after the recruitment of RNAse H. As the paternal copy of *UBE3A* is no longer repressed, the paternal UBE3A protein is produced, thereby restoring UBE3A function (Figure 3). The ASO appears to be well tolerated by mice. This strategy is promising and original. Similar developments in humans should be possible (Meng et al., 2015).

### 1.4 Why should ASOs be preferred over conventional treatment

#### 1.4.1 Route of administration and tissue biodistribution

The best route of administration for an ASO is the one enabling it to reach its target. Indeed, the pharmacological profile of ASOs is largely influenced by their protein‐binding capacity. These interactions depend principally on the chemical structure of the ASOs. ASO‐PS have a high plasma protein‐binding capacity (>90%), resulting in wide systemic distribution and possible uptake by target tissues and cells. Unfortunately, these interactions also contribute to the toxicity of ASOs. Chemical modifications designed to minimize these effects and increase the therapeutic index are currently being studied (Shen et al., 2019; Wang, Han, Sun, Chen, & Chen, 2019). ASO‐PNA and ASO‐MF, which are uncharged, are rapidly excreted in urine, limiting their tissue absorption. The coupling of these molecules to peptides (PPMO) increases their tissue biodistribution, as previously suggested (Jearawiriyapaisarn, Moulton, Sazani, Kole, & Willis, 2010).

Intravenous (iv) administration should be favored for systemic effects with the most recent generation of ASOs. This administration route maximizes the bioavailability of ASOs and leads to rapid distribution to highly irrigated organs (liver, kidney, spleen). By contrast, distribution is weak in the lungs, cardiac muscle, and skin, and negligible in adipose tissue. Nonetheless, iv administration is considered to be the optimal route for the treatment of Duchenne muscular dystrophy (Exondis 51, as highlighted below) (Mendell et al., 2016) and modifications of these ASOs are currently being studied to optimize their biodistribution to the muscles through coupling to a fatty acid (Prakash et al., 2019) or peptide (Pip‐6 PMO) (Figure 3) (Betts et al., 2012).

The subcutaneous (sc) route is an interesting alternative to the iv route, increasing the ease of administration, when possible. Inotersen (Tegsedi®), marketed by Ionis Pharmaceuticals and Akcea Therapeutics, has recently been licensed for sc administration in the United States, Canada, and Europe (Figure 2) (Keam, 2018). This drug is indicated for the treatment of hereditary transthyretin amyloidosis (hATTR). This disease is caused by an autosomal‐dominant mutation of the transthyretin (*TTR*) gene. Approximately 100 mutations of this gene have been described, associated with varying degrees of disease severity due to amyloid deposits in tissues and nerves. Such deposits lead to the progressive onset of cardiomyopathy, nephropathy, polyneuropathy, and gastrointestinal disorders (Connors, Lim, Prokaeva, Roskens, & Costello, 2003). As the liver is the principal organ that produces TTR, the standard treatment for hATTR is liver transplantation. However, in certain patients, the disease progresses to other organs, despite liver transplantation (Liepnieks, Zhang, & Benson, 2010). Inotersen is a second‐generation 2′‐*O*‐methoxyethyl‐modified ASO that inhibits liver synthesis of TTR. In clinical trials, this ASO has been shown to improve the quality of life of patients relative to placebo, regardless of the causal mutation and disease stage. Inotersen is administered subcutaneously and may be self‐administered after instruction by healthcare staff (Benson et al., 2018). When sc administration is possible, ASOs offer a targeted solution, while minimizing the constraints of administration.

In parallel to systemic administration, ASOs can be administered locally, allowing direct and local targeting. Local administration can also reach compartments not accessible by iv or sc routes. This is true, in particular, for the administration of formivirsen (Vitravene®) to the eye (Vitravene Study Group, 2002). Intrathecal administration is also an interesting alternative route of administration for the central nervous system, as the blood–brain barrier is impermeable to ASOs. Therefore, only minute amounts of ASOs reach the brain after iv or sc administration (Phillips et al., 1997). Intrathecal administration results in significant bioavailability in the brain and spinal cord. The invasive nature of this route limits its use but it is still the only way ASOs can be used to treat neurological diseases. It allows direct targeting of the tissue, without systemic exposure, a major source of toxicity. It also avoids renal and hepatic elimination, resulting in local maintenance of the concentration of the molecule. These factors all make ASOs good candidates for the treatment of neurological diseases. Many molecules are currently in clinical trials, at various stages of evaluation, to obtain marketing authorization (Rinaldi & Wood, 2017). For example, Huntington's disease, like DM1, is a triplet‐repeat disease, affecting the *huntingtin* gene. In the presence of abnormally expanded CAG repeats, toxic accumulation of the protein occurs (Ambrose et al., 1994), causing motor and cognitive disorders (Lawrence et al., 1996). The number of repeats correlates with age at the onset of symptoms, but not disease severity or progression (Duyao et al., 1993). ASOs that catalyze RNAse H‐mediated degradation of *huntingtin* mRNA decrease the production of the protein. Transient ASO infusion into the CNS therefore limits the progression of the disease in a mouse model and primates (Kordasiewicz et al., 2012).

ASOs can also be locally delivered by inhalation (nebulization). This is true for eluforsen in cystic fibrosis patients homozygous for the F508del‐CFTR mutation (cystic fibrosis transmembrane conductance regulator). This molecule restores the activity of the CFTR channel and, therefore, the respiratory function of treated patients (Drevinek et al., 2019; Sermet‐Gaudelus et al., 2019). This route of administration is not invasive and self‐administration should be possible.

Another very promising option to increase specific uptake independently of the route of administration is the conjugation of specific molecules to ASOs as mentioned previously (fatty acid, peptides or *N*‐acetylgalactosamine) (reviewed in Benizri et al., 2019). One of the best examples thus far developed is the conjugation of an *N*‐acetylgalactosamine (GalNac) moiety to the ASO. The GalNAc receptor ligand specifically binds to the Asialoglycoprotein Receptor (ASGR), which is highly expressed at the surface of hepatocytes, substantially increasing the targeting of GalNac‐ASO to the liver and its uptake (Tanowitz et al., 2017). The conjugation of triantennary *N*‐acetylgalactosamine (GalNAc3) makes ASOs up to 30 times more potent non‐conjugated ASOs, supporting lower doses and increased safety (Crooke et al., 2019; Prakash et al., 2014). These ASOs thus hold great therapeutic potential for hepatic diseases, such as hepatocellular carcinoma among others (Y. Kim, Hu, et al., 2019). Indeed, several GalNac‐conjugated oligonucleotides are undergoing pivotal clinical investigation (reviewed in Y. Huang, 2017). Very recently, a phase 2a trial, testing a GalNac3‐conjugated ASO (referred as APO(a)‐L_RX_) that targets lipoprotein(a) mRNA (*LPA*), showed a significant dose‐dependent reduction in lipoprotein(a) levels in patients with cardiovascular disease (ClinicalTrials.gov number, NCT03070782). This is a major breakthrough, as APO(a)‐L_RX_ is the first and currently only drug in clinical development designed to selectively and robustly inhibit the production of Lp(a) (Tsimikas et al., 2020).

#### 1.4.2 Orphan genetic diseases

There is currently no cure for most orphan diseases. Antisense approaches would be a great asset for such diseases, as shown by the development of treatments for Duchenne muscular dystrophy (DMD) and very recently for neuronal ceroid lipofuscinosis 7 (CLN7).

DMD is a rare genetic disorder linked to the X chromosome. It affects between 1 in 3,500 and 1 in 6,000 newborn boys worldwide each year (Bushby et al., 2010; Emery, 1991). It is caused by nonsense mutations of the dystrophin gene. These deleterious mutations (deletions, insertions, and point mutations, resulting in a frameshift and the appearance of a premature stop codon) lead to the complete absence of dystrophin. Mutations have been found in all exons, but exons 47 to 63 are the most frequently affected (Flanigan et al., 2009).

Dystrophin is a structural protein of striated muscles that plays an essential role in maintaining muscle integrity. Defective dystrophin production has dramatic consequences for the patient. Indeed, children and young adults with DMD progressively develop severe neuromuscular deficiencies. They lose the ability to walk, and then suffer cardiorespiratory problems and damage to the muscles of the digestive tract. All these symptoms lead to the premature death of DMD patients (Brooke et al., 1989). There are other forms of dystrophy that are generally less severe than DMD, such as Becker's dystrophy (Muntoni, Torelli, & Ferlini, 2003). The Duchenne and Becker dystrophies have the same origin—alterations to the Xp21 locus—but the mutations found in Becker's dystrophy are less deleterious. The produced altered dystrophin protein remains partially functional (Bushby et al., 1993; Monaco, Bertelson, Liechti‐Gallati, Moser, & Kunkel, 1988). The symptoms observed in patients with Becker dystrophy are highly heterogeneous. The most severely affected patients have symptoms similar to those seen in patients with DMD, but some patients are almost asymptomatic. Despite many years of research on these diseases, they remain incurable. Current treatments aim to slow disease progression and to preserve as many muscle fibers as possible.

The much more severe clinical presentation of DMD relative to Becker dystrophy led to the design of ASOs to generate a Becker‐like phenotype in DMD patients. By forcing the exclusion of the exon carrying the deleterious mutation, it is possible to generate a partially functional truncated protein. In this situation, ASOs bind to the pre‐mRNA at the splice site of the mutated exon. Several studies have been conducted to select candidate exons for exclusion (Aartsma‐Rus et al., 2004; J. C. van Deutekom et al., 2007; J. C. T. van Deutekom et al., 2001). Several molecules are currently in clinical trials, including eteplirsen, marketed under the name Exondys 51® by Sarepta Therapeutics. After debates concerning its efficacy, eteplirsen was finally approved by the FDA in 2016. It is a third‐generation ASO, a phosphorodiamidate morpholino oligomer (PMO), which does not allow RNAse H activity. It hybridizes to the exon 51 splice site, leading to exon 51 skipping. Eteplirsen treatment simulates a milder form of dystrophy in the 13% of DMD patients carrying a deleterious mutation of exon 51. This ASO was injected (iv) once per week. After 36 months, eteplirsen‐treated patients showed a lower incidence of loss of ambulation (Mendell et al., 2016). There are other ASOs targeting other exons that work in a similar manner. SRP‐4053 (Golodirsen) targets exon 53 and SRP‐4045 (ClinicalTrials.gov Identifier: NCT02500381) and PRO044 (ClinicalTrials.gov Identifier: NCT01037309) target exon 45 (Ferlini et al., 2013). Although the targeted exons are different, the therapeutic strategy remains the same, and comparable development highlights the flexibility offered by the use of ASOs. These antisense therapies are particularly effective for decreasing clinical signs in skeletal muscle but have a limited effect on heart disease. Indeed, the heart is poorly irrigated, limiting the biodistribution of PMOs. New molecules, such as the antisense Pip6a‐PMO, as previously mentioned, are being studied to target heart muscle. This molecule consists of an ASO coupled to peptides (peptide‐conjugated phosphorodiamidate morpholino oligomer (PPMO)). The peptide domain, rich in cationic amino acids (including arginine), adds a charge to the initially uncharged PMO, thereby increasing its bioavailability. These new molecules have been administered by the iv route to mice in a model of DMD. The preliminary results indicate an improvement in dystrophin production in the muscles, including the heart, through the same mode of action: exon skipping (Blain et al., 2018).

Other innovative and targeted therapies are also being tested. They involve the use of exogenous synthetic dystrophin produced by a modified adenovirus (AAV). This strategy is, however, limited by the intrinsic packaging capacity of these viruses, which can hold, at most, only one third of the dystrophin sequence. Recent studies have been conducted with lentiviruses (Counsell et al., 2017). In parallel, cell therapies involving the use of stem cells to promote cell regeneration are also being investigated (reviewed Sienkiewicz, Okurowska Zawada, Paszko Patej, Kawnik, & Kulak, 2015). Although promising, these strategies are still experimental. It is hoped that it will be possible to use them to treat DMD patients. They will probably be used with ASOs to improve their efficacy (Figure 3).

Very recently, outstanding results were obtained with a tailor‐made ASO, developed in record time and specifically designed for a single patient with neuronal ceroid lipofuscinosis 7 (CLN7), a form of Batten's disease (Y. Kim, Hu, et al., 2019). This is a rare autosomal recessive disorder that affects the nervous system, with the mean age of patients at onset of 3.3 years and a fatal outcome. Genetic testing of the patient revealed a single pathogenic heterozygous mutation in the *CLN7* gene (also known as *MFSD8*) and an SVA (SINE‐VNTR‐ALU) retrotransposon insertion in the second allele. The SVA insertion affects splicing of the nearby gene, inducing mis‐splicing of exon 6 of *CLN7*. 22‐nucleotide ASOs (phosphorothioate and 2′‐*O*‐methoxyethyl, same backbone and sugar chemistry modifications as nusinersen) were designed and tested in patient fibroblasts to restore the altered splicing. The most effective were retained for further development. This patient‐customized ASO was named Milasen. The urgency of the patient's clinical state sped up toxicological evaluation. After obtaining FDA authorization and institutional review board approval, the investigators administered the compound intrathecally to the patient in ascending doses, as for nusinersen, which had been safely administrated to infants with SMA (Finkel et al., 2017). There were no serious adverse events during the first year of treatment. The efficacy of the treatment was associated with an objective reduction in seizures (J. Kim, Jo, et al., 2019). This study strongly demonstrates how ASOs can be used as a component of individualized genomic medicine (Figure 2).

#### 1.4.3 Cancer

Targeted therapies have revolutionized cancer management by making it possible to specifically target the genetic abnormalities that cause or play a major role in tumor development. Major therapeutic successes have been obtained with inhibitors that specifically target constitutively active tyrosine kinases, such as specific inhibitors of the constitutively active form of the BRAF kinase (BRAF‐V600) in BRAF‐V600 mutated metastatic melanoma (Chapman et al., 2011) The use of blocking antibodies specific for an oncogenic pathway has also resulted in therapeutic success (anti‐EGFR antibody in metastatic colon cancer and anti‐HER2 in breast cancer with HER‐2 amplification) (Li et al., 2017; Ross et al., 2009). ASOs are particularly interesting and appropriate tools in cancer treatment because of the versatility of their action and the possibility of generating ASOs specific for target sequences. As previously mentioned, they can be used not only to decrease the expression of coding oncogenic drivers but can also target non‐coding RNAs. However, despite several attempts and ongoing trials, none of these compounds have yet obtained marketing authorization for use in oncology. Several ASOs targeting key oncogenic actors are currently in clinical trials (phase I‐III).

Danvatirsen (AZD9150 and IONOS‐STAT3‐2.5) was designed to reduce the production of STAT3 (signal transducer and activator of transcription 3). STAT3 plays a key role in transducing signals that promote cell growth and survival, and is thus a potential drug target for several types of cancer (hematological, lung, breast, colorectal, etc.) (Hong et al., 2015; Reilley et al., 2018). Furthermore, the combination of AZD9150 with conventional chemotherapy for the treatment of neuroblastoma decreases tumorigenicity and increases chemosensitivity (Odate et al., 2017). Other trials of combinations of AZD9150 with immunotherapy (anti‐PD‐L1), with or without conventional chemotherapy, are also underway (phase II trials, NCT029883578, NCT03421353).

Trabedersen (OT‐101), a phosphorothioate ASO, targets the transforming growth factor beta (TGF‐β) pathway. Trabedersen specifically targets TGF‐β2, which plays a key role in the late stages of tumor development (epithelial‐mesenchymal transition, EMT). Several phase I/II clinical trials have evaluated the utility of this ASO to treat high‐grade gliomas. Although unsuccessful, these trials made it possible to evaluate the safety of this ASO and its administration. Promising results have also been obtained for the treatment of advanced pancreatic cancer (reviewed in Takakura et al., 2019).

Custirsen (OGX‐011), a second‐generation ASO, targets the clusterin mRNA. Clusterin is a stress‐dependent chaperone protein. Its anti‐apoptotic activity promotes the development of resistance to anticancer treatments. Clusterin inhibition therefore potentiates the therapeutic activity of conventional chemotherapies (Zhang, Ge, Zhu, & Salaita, 2014). Unlike the previous examples, Custirsen would therefore render the tumors of patients more sensitive to chemotherapy. However, it yielded no significant improvement in overall survival in a phase III trial on metastatic prostate cancer when used in combination with conventional first‐line treatment (docetaxel [Taxotere®] and prednisolone). Nevertheless, a *post‐hoc* analysis highlighted an improvement in overall survival for a subgroup of patients with a poor prognosis (Chi et al., 2017).

Apatorsen (OGX‐427), a second‐generation ASO, targets another chaperone protein, heat‐shock protein 27 (Hsp27). Hsp27 levels are very high in some cancers and this is associated with a poor prognosis. The inhibition of Hsp27 decreases tumor growth and sensitizes the tumor to cytotoxic chemotherapies. The preliminary results of a phase II trial assessing docetaxel alone or in combination with apatorsen for the treatment of metastatic urothelial carcinoma showed a slight increase in overall survival in the combination group. These results are promising and require confirmation (Rosenberg et al., 2019).

Many developments are still at the preclinical stage. Original ASO‐based treatments have been proposed for very aggressive cancers for which there are currently few or no effective strategies. For example, the group of Obika recently proposed an original therapeutic strategy to restore the activity of a tumor suppressor in small‐cell lung cancer (Rudin et al., 2019; Shimojo et al., 2019). Molecular data showed an increase in the expression of the SR/SR4 splicing regulator (Ser/Arg repetitive matrix 4), causing abnormal splicing of the tumor suppressor REST (RE1‐silencing transcription factor) (Westbrook et al., 2005). The non‐active, spliced form competes with the normal form and inhibits its tumor suppressor activity (Shimojo, Paquette, Anderson, & Hersh, 1999). A gapmer: LNA‐type ASO specifically targeting the SRRM4 splice regulator abolished or decreased the inappropriate splicing of the REST tumor suppressor. This ASO, which was shown to be effective in vivo (mouse model), is of therapeutic interest, particularly as its effectiveness can be monitored with a plasma marker (Shimojo et al., 2019).

In parallel to these approaches aiming to modify the expression of oncogenic coding molecules, several ASOs have been designed to target non‐coding RNAs. In particular, lnc‐RNAs, and miRNAs have emerged as important regulators of the various phases controlling tumor transformation and progression. These non‐coding RNAs are, therefore, prime targets for ASOs (Huarte, 2015; Ling, Fabbri, & Calin, 2013; Rupaimoole & Slack, 2017).

The Lnc‐RNAs of interest include *MALAT‐1* (metastasis‐associated lung adenocarcinoma transcript 1). Decreasing *MALAT‐1* levels with an ASO significantly inhibits tumor growth and metastasis in lung and breast cancers (Arun et al., 2016; Gutschner et al., 2013).

The *SAMMSON* pro‐tumoral lnc‐RNA is co‐amplified with the melanocyte‐specific *MITF* gene in 10% of metastatic melanomas. Importantly, 90% of melanomas specifically express *SAMMSON*, which is not expressed in healthy cells. *SAMMSON* is thus a specific target of interest. The pro‐tumor activity of *SAMMSON* is based on its interaction with the p32 protein, a major regulator of mitochondrial activity and metabolism. The use of a *SAMMSON*‐specific gapmer reduced the clonogenicity of cells expressing *SAMMSON*, regardless of the genetic status of the tumor (mutations of *BRAF*, *NRAS*, *TP53*). In vivo, the potential therapeutic value of these gapmers has been demonstrated in PDX models (patient‐derived xenografts) (Figure 2) (Leucci et al., 2016).

**Figure 4:**
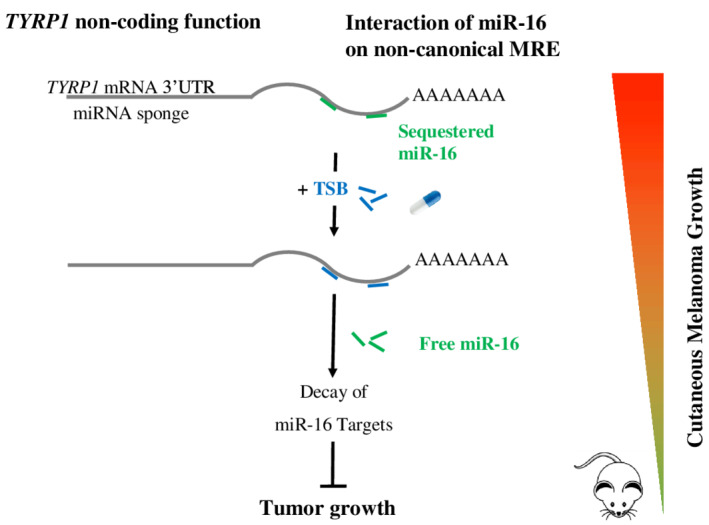
Inhibition of *TYRP1* miRNA sponge activity as a therapeutic strategy

Our group also developed an original antisense strategy for the treatment of metastatic melanoma following the discovery of a novel mechanism of miRNA sequestration (Figure 2) (Gilot et al., 2017; Migault, Donnou‐Fournet, Galibert, & Gilot, 2017). As previously described, the canonical silencing activity of miRNAs requires perfect base pairing between the seed region (2nd to 7th nucleotide) of the miRNA and that of the MRE on the mRNA (Bartel, 2004). The base complementarity between the miRNA and mRNA can, however, be imperfect, leading to non‐canonical interactions. These interactions lack miRNA silencing activity. Our work is based on the identification of non‐canonical interactions between miR‐16 and the *tyrosinase‐related protein 1* (*TYRP1*) mRNA. *TYRP1* is specifically expressed in the melanocyte lineage and is highly expressed in melanoma. We identified three non‐canonical MREs for miR‐16 in the 3′ UTR of *TYRP1*. These non‐canonical MRE‐16s provide a non‐coding function to *TYRP‐1* mRNA, which acts as a miRNA‐sponge. Indeed, such non‐canonical binding results in the sequestration of miR‐16, with delayed or no *TYRP1* mRNA degradation. As miR‐16 has tumor suppressor activity in cutaneous melanoma, its sequestration promotes tumor growth. In this context, we designed ASOs to prevent such sequestration. They consist of target‐site blockers (TSB): a third‐generation ASO, consisting of a mixmer of DNA and LNA, with no RNAse H activity. These TSBs were designed to be specific for *TYRP1* mRNA, hybridizing to *TYRP1* in place of miR‐16. As they are perfectly complementary to the *TYRP1* mRNA along their entire length, their affinity for *TYRP1* mRNA is greater than that of miR‐16. TSB displaces miR‐16, allowing the redirection of miR‐16 to other targets. The tumor suppressor activity of miR‐16 is thus restored. These TSBs were shown to be effective in vivo in a PDX model. This specific and ground‐breaking treatment strategy can restore the activity of miR‐16 exclusively in cells expressing *TYRP1*. This innovation circumvents the need for a synthetic microRNA, which through its systemic effects, would have consequences throughout the body (Figures 3 and 4).

## 2 CONCLUSION AND PERSPECTIVES

Improvements in molecular knowledge and understanding the mechanisms underlying disease‐development has made possible the shift from therapeutic approaches towards personalized medicine. At the same time, high throughput sequencing has made it possible to perform powerful molecular diagnoses required for the characterization of gene alterations and the implementation of targeted therapies. Together, these approaches have led to the refinement of diagnoses and prognostic analyses for patients and their diseases, and selection of the patients most likely to respond to treatment.

In this context, ASOs are powerful tools that directly target RNA. Through a non‐exhaustive set of pioneering examples, we show here that ASOs can be used to treat many diseases caused by diverse genetic alterations and molecular programs. These diseases include neurodegenerative disorders and cancer, both major public health issues, as rare diseases currently lacking effective treatment. ASOs offer great flexibility of use, targeting coding and non‐coding RNAs, with diverse mechanism of action. They may act as splicing modulators, increase or diminish RNA and protein levels, and mask or disrupt miRNA and protein sequestration. Indeed, their chemistry can be adapted to modulate their effect (w/ or w/o RNAse H activity) and bioavailability and, importantly, their sequence can be adapted to genetic alterations. The fact that ASO can be customized in a sequence‐specific fashion to match patient disorders was recently strongly underscored with the splice‐switch ASO Milasen (J. Kim, Jo, et al., 2019). This example of successful drug‐development and treatment within 1 year after first contact with the patient is probably the most advanced example of personalized medicine. Such spectacular development is still experimental and may require further exploration with careful examination of clinical and ethical issues.

Important chemical developments have been made to diminish the toxicity of ASOs, and the most representative molecules of these chemical classes are well tolerated. However, substantial toxicity may still be observed when they are used chronically. This may be an important drawback that should be lessened by the development of conjugated‐ASOs that promote cell‐specific uptake.

ASOs may be used in monotherapy or in combination with conventional approaches. They are therefore both an alternative for diseases with no effective available treatments and an option for use in patients with refractory diseases, as second‐ or third‐line treatments.

In conclusion, the phrases “Targeting the Untargetable” and “Treating the Untreatable” summarize the potential of ASO strategies, thus revolutionizing personalized medicine.

## AUTHOR CONTRIBUTIONS

**Anais M. Quemener:** Conceptualization; writing‐review and editing. **Laura Bachelot:** Conceptualization; writing‐review and editing. **Anne Forestier:** Writing‐review and editing. **Emmanuelle Donnou‐Fournet:** Writing‐review and editing. **David Gilot:** Writing‐review and editing. **Marie‐Dominique Galibert:** Conceptualization; funding acquisition; writing‐review and editing.

## CONFLICT OF INTEREST

The authors have declared no conflicts of interest for this article.

## RELATED WIREs ARTICLES

Natural antisense transcripts in diseases: From modes of action to targeted therapies

Alternative‐splicing defects in cancer: Splicing regulators and their downstream targets, guiding the way to novel cancer therapeutics

Splicing and neurodegeneration: Insights and mechanisms
